# Clinical characteristics and risk factors for perinatal arterial ischemic and hemorrhagic stroke: a comparative study

**DOI:** 10.3389/fped.2025.1540173

**Published:** 2025-07-01

**Authors:** Chongchong Liu, Yi Zhang, Run Yang, Shiwen Xia

**Affiliations:** ^1^ Department of Neonatology, Maternal and Child Health Hospital of Hubei Province, Tongji Medical College, Huazhong University of Science and Technology, Wuhan, China; ^2^ Department of Pediatric Neurology, Maternal and Child Health Hospital of Hubei Province, Tongji Medical College, Huazhong University of Science and Technology, Wuhan, China

**Keywords:** ischemic stroke, hemorrhagic stroke, risk factors, cesarean delivery, thrombocytopenia

## Abstract

**Background:**

Perinatal cerebral infarction (PCI) is a common cause of neurological complications in neonates. This study aimed to compare the clinical characteristics and risk factors of perinatal arterial ischemic stroke (PAIS) and perinatal hemorrhagic stroke (PHS) to inform early recognition and intervention strategies.

**Methods:**

We conducted a retrospective analysis of 124 neonates diagnosed with PCI, admitted to the neonatal intensive care unit (NICU) between June 2015 and December 2023. The neonates were divided into two groups—PAIS and PHS—based on clinical symptoms and cranial imaging findings.

**Results:**

Of the 124 patients, 87 (70.2%) were diagnosed with PAIS, while 37 (29.8%) had PHS. Clonic seizures were observed in 78 cases (62.9%), with apnoea noted in 11 cases (12.6%) within the PAIS group and jaundice in 8 cases (21.6%) within the PHS group. Lesions were primarily located in the left cerebral hemisphere in 53 cases (41.4%), whereas PHS lesions frequently involved the thalamus and basal ganglia (12 cases, 32.4%). Statistical analysis revealed significant differences in risk factors between the PAIS and PHS groups. The PAIS group had a higher rate of conversion from failed trial of labor to cesarean section compared to the PHS group (*P* = 0.012). Additionally, postnatal thrombocytopenia was more commonly associated with the PHS group than the PAIS group (*P* = 0.034).

**Conclusions:**

Our findings indicate that PAIS is more prevalent within the studied population, with a notable correlation between failed labor trials resulting in cesarean sections and the incidence of PAIS. This suggests a potential link between complications during labor and the occurrence of ischemic strokes. In contrast, postnatal thrombocytopenia was found to be significantly more common in the PHS group, indicating a possible association between low platelet counts and hemorrhagic strokes.

## Introduction

Perinatal cerebral infarction (PCI), commonly referred to as perinatal stroke, is characterized by localized brain damage in neonates occurring within the first 28 days after birth due to cerebrovascular disease (1). PCI encompasses two primary categories: perinatal ischemic stroke (PIS) and perinatal hemorrhagic stroke (PHS). PIS is defined as “a heterogeneous group of disorders occurring between the 20th week of fetal life and the 28th day post-birth, confirmed by neuroimaging or neuropathological studies, secondary to arterial or cerebral venous thrombosis or embolism leading to focal cerebral blood flow interruption” (2). Within PIS, there are two subtypes: perinatal arterial ischemic stroke (PAIS) and cerebral sinovenous thrombosis (CSVT). In contrast, PHS refers to intracranial hemorrhagic lesions that can occur within the ventricles, brain parenchyma, or subarachnoid space. Hemorrhagic infarction can be classified into two types: primary hemorrhage resulting from vascular anomalies or hemorrhagic diseases, and secondary transformation due to arterial or venous ischemic stroke (3).

The incidence of PCI varies across populations, with international literature estimating a prevalence of approximately 1 in 1,600–3,000 live births (1). This rate exceeds the risk of stroke in children by a factor of ten (4) and is significantly higher than the incidence of pediatric cerebral infarction. The estimated incidence rate of PAIS ranges from 17.8 to 35 cases per 100,000 live births (5), accounting for approximately 71% of all perinatal strokes (6). CSVT, on the other hand, is a rare cause of stroke, with reported incidence rates in neonates and children of about 0.67 per 100,000 (7). PCI can lead to various sequelae, including motor disorders, cognitive impairments, cerebral palsy, and epilepsy, which profoundly affect quality of life and impose a considerable economic burden on healthcare systems. Consequently, PCI is recognized as a major contributor to chronic neurological disabilities in children (8, 9).

Ischemic stroke represents the most prevalent form of stroke; therefore, previous research has predominantly focused on identifying risk factors associated with arterial ischemic stroke, while less attention has been given to the risk factors related to hemorrhagic stroke. Furthermore, there has been a lack of comprehensive analysis comparing the risk factors between these two types of strokes. To address this gap, our study collected demographic baseline data, clinical manifestations, and cranial imaging characteristics of neonates diagnosed with PCI. We categorized the subjects based on cranial magnetic resonance imaging (MRI) findings into PAIS and PHS groups. Analyzing risk factors and prognostic differences between these groups aims to enhance clinical diagnosis, treatment strategies, and prognostic assessment.

## Materials and methods

### Study subjects

This study involved a retrospective analysis of the clinical data of 124 neonates diagnosed with PAIS and PHS who were admitted to the Neonatal Intensive Care Unit at Hubei Women and Children's Hospital between June 2015 and December 2023. The study was approved by the Medical Ethics Committee of Maternal and Child Health Hospital of Hubei Province (approval number: 2024-023-01), which also granted a waiver for informed consent.

### Inclusion and exclusion criteria

Inclusion criteria: (1) age less than 28 days; (2) meets the definition of PCI: (a) clinical manifestations: any neurological symptoms (including seizures, neurological deficits, reduced responsiveness, cyanosis, recurrent apnea, and severe jaundice); (b) neuroimaging [e.g., MRI or computed tomography (CT)] findings (Perinatal lesions): (i) arterial ischemia: partial or complete occlusion of a cerebral artery associated with focal brain damage, where the pattern of brain injury can only be explained by the occlusion of specific cerebral arteries; (ii) hemorrhage: defined as intracranial hemorrhage within the ventricles, brain parenchyma, or subarachnoid space (10).

Exclusion criteria: (1) neuroimaging (e.g., MRI or CT) showing venous thrombosis: defined as the presence of thrombus in cerebral veins or venous sinuses, and partial or complete occlusion; (2) hemorrhage caused by cranial injury, such as epidural, subdural, cranial fracture, or subdural hemorrhage; (3) systemic hypoxic-ischemic injury; (4) intracranial lesions caused by genetic metabolic diseases; (5) congenital cerebrovascular anomalies.

### Research methods

Medical records were reviewed to collect and organize the following indicators: gestational age, sex, birth weight, multiple births (twins or more), maternal medical history (such as gestational hypertension and diabetes), family history (maternal autoimmune diseases and familial thrombotic diseases), perinatal infection (including premature rupture of membranes >18 h), delivery conditions, amniotic fluid contamination, placental abnormalities (including placenta previa, placental abruption, placental calcification, velamentous placenta), umbilical cord abnormalities (including nuchal cord >1 loop, twisted umbilical cord, true knot of umbilical cord, single umbilical artery, thin umbilical cord), intrauterine distress, postnatal asphyxia (Apgar score <7 at 5 min), intrauterine growth retardation, small for gestational age infants, cardiac echocardiography, infection markers, platelet count (<100 × 10^9^/L), coagulation function, initial symptoms, hospital stay duration, cranial imaging, electroencephalogram results, MRI findings, and follow-up outcomes of the children.

### Methods of EEG monitoring and definition of abnormal EEG

Methods of EEG monitoring: multichannel video EEG was recorded using a Nicolet monitor (Carefusion NeuroCare, Wisconsin, USA) with a 10–20 conductive electrode placement system modified for neonates. Scalp electrodes were placed at positions F3, F4, C3, C4, T3, T4, O1, O2, Cz, P3, and P4, fixed within an elastic cap, and EEG activity was recorded in frontal, central, temporal, parietal, and occipital regions, with impedance maintained at less than 5 kΩ, and the monitoring was labeled for various sleep states and events. The EEG monitoring lasted for at least 6 h, with sleep duration including at least one complete sleep cycle, and all EEGs were read by a person qualified in EEG. Classification: into electro-clinical (with clinical sign), electrographic only (without clinical signs) and unclassified seizure type. Electro-clinical (with clinical sign) can have a motor (automatisms, clonic, epileptic spasms, myoclonic, tonic), non-motor (autonomic, behavior arrest), or sequential presentation (11). Defined an electrographic neonatal seizure: a sudden, abnormal EEG event, defined by a repetitive and evolving pattern with a minimum 2 μV peak-to-peak voltage and duration of at least 10 s (12).

### Data collection

We conducted a search of the electronic medical records database at Hubei Women and Children's Hospital for cases diagnosed with “cerebral embolism,” “cerebral infarction,” “intracranial hemorrhage,” “subarachnoid hemorrhage,” “intracranial hematoma,” and “intraventricular hemorrhage” from June 2015 to December 2023. A total of 389 cases were identified; after applying the exclusion criteria, 265 cases were eliminated, resulting in a final sample of 124 cases. The imaging studies for all pediatric patients were initially evaluated by experienced pediatric neuroradiologists and subsequently reviewed by pediatric neurologists to confirm the characteristics of the infarction, distinguish between ischemic and hemorrhagic strokes, and identify the affected vessels and locations of the lesions.

### Statistical analysis

Statistical analysis was performed using SPSS version 27.0. The Kolmogorov–Smirnov test was used to determine data normality. Normally distributed quantitative data were expressed as mean ± standard deviation $\left(\bar{x}\pm s\right)$. Non-normally distributed quantitative data were presented as median and interquartile range. To compare non-normally distributed quantitative data between groups, the Wilcoxon rank-sum test was employed. For categorical variables, frequencies and percentages were calculated, and the chi-square test was utilized for comparisons. A *P*-value of less than 0.05 was deemed statistically significant.

## Results

### General data

The study included a total of 124 neonates diagnosed with cerebral infarction, comprising 87 cases (70.2%) in the PAIS group and 37 cases (29.8%) in the PHS group. The average gestational age was 38.8 ± 1.8 weeks (ranging from 31.3 to 42 weeks), with 110 term infants and 14 preterm infants. The average birth weight was 3248 ± 542 g (with a range of 1270–4450 g). The average maternal age was 29.23 ± 4.64 years, and there were 5 sets of twins among the cases. A comparison of general data between the PAIS and PHS groups revealed no significant differences in demographic characteristics (Table 1).

**Table 1:** Comparison of general data between the PAIS and PHS groups.

Variable	PAIS group (*n* = 87)	PHS group (*n* = 37)	*χ*^2^/*Z* value	OR (95% CI)	*P*-value
Gestational age [M (P25, P75), weeks]	39.1 (38.2, 40.1)	39.4 (37.1, 40.0)	0.238	—	0.812
Birth weight (g)	3,247 ± 544	3,250 ± 545	0.003	—	0.998
Mother's age [M (P25, P75), Y]	29 (26.0, 32.0)	29 (26.5, 31.0)	0.123	—	0.902
Male infants [%]	59 (67.8)	24 (64.9)	0.102	1.141 (0.507–2.568)	0.749

PAIS, perinatal arterial ischemic stroke; PHS, perinatal hemorrhagic stroke; OR, odds ratio. $\bar{x}\pm s$.

### Prenatal, perinatal, and postnatal risk factors

Prenatal and perinatal risk factors: Intrauterine distress was noted in 28 cases, with 19 cases (21.8%) in the PAIS group and 9 cases (24.3%) in the PHS group. Amniotic fluid contamination by meconium occurred in 30 cases, including 20 cases (23%) in the PAIS group and 10 cases (27%) in the PHS group. Placental abnormalities were present in 11 cases, with 8 cases (9.2%) in the PAIS group and 3 cases (8.1%) in the PHS group. Umbilical cord abnormalities affected 30 cases, with 21 cases (24.1%) in the PAIS group and 9 cases (24.3%) in the PHS group. Small for gestational age infants were identified in 14 cases, with 11 cases (12.6%) in the PAIS group and 3 cases (8.1%) in the PHS group. Postnatal asphyxia occurred in 19 cases, with 11 cases (12.6%) in the PAIS group and 8 cases (21.6%) in the PHS group. Among the neonates studied, only 2 presented with intrauterine growth restriction, and just 1 had a maternal history of systemic lupus erythematosus. A history of eclampsia/pre-eclampsia was noted in 12 cases (9.7%), with 10 cases (11.5%) in the PAIS group and 2 cases (5.4%) in the PHS group. Gestational diabetes was reported in 9 cases (7.3%), with 8 cases (9.2%) in the PAIS group and 1 case (2.7%) in the PHS group. Prenatal infections were found in 35 cases (28.2%), with 22 cases (25.3%) in the PAIS group and 13 cases (35.1%) in the PHS group. Of the total neonates, 65 (52.4%) underwent cesarean sections following a failed trial of labor, including 52 cases (59.8%) from the PAIS group and 13 cases (35.1%) from the PHS group (Table 2).

**Table 2:** Univariate comparison between PAIS and PHS.

Variable	PAIS group (*n* = 87)	PHS group (*n* = 37)	*χ*^2^/*Z* value	OR (95% CI)	*P*-value
Small for gestational age [*n* (%)]	11 (12.6)	3 (8.1)	0.533	0.610 (0.160–2.326)	0.465
Maternal eclampsia/pre-eclampsia [*n* (%)]	10 (11.5)	2 (5.4)	1.101	0.440 (0.092–2.115)	0.294
Maternal gestational diabetes [*n* (%)]	8 (9.2)	1 (2.7)	1.626	0.274 (0.033–2.276)	0.202
Maternal perinatal infection [*n* (%)]	22 (25.3)	13 (35.1)	1.243	1.600 (0.698–3.671)	0.265
Failed trial labor to cesarean [*n* (%)]	52 (59.8)	13 (35.1)	6.317	0.366 (0.164–0.811)	0.012*
Meconium-stained amniotic fluid [*n* (%)]	20 (23)	10 (27)	0.231	1.241 (0.514–2.994)	0.631
Placental abnormalities [*n* (%)]	8 (9.2)	3 (8.1)	0.038	0.871 (0.218–3.486)	0.846
Umbilical cord abnormalities [*n* (%)]	21 (24.1)	9 (24.3)	0.000	1.010 (0.412–2.478)	0.982
Intrauterine distress [*n* (%)]	19 (21.8)	9 (24.3)	0.092	1.150 (0.464–2.849)	0.762
Postnatal asphyxia [*n* (%)]	11 (12.6)	8 (21.6)	1.613	1.906 (0.697–5.213)	0.204
Postnatal infection [*n* (%)]	36 (41.4)	15 (40.5)	0.008	0.966 (0.442–2.113)	0.931
Hypercoagulable state [*n* (%)]	7 (8.0)	2 (5.4)	0.269	0.653 (0.129–3.303)	0.604
Congenital heart disease [*n* (%)]	30 (34.5)	9 (24.3)	1.243	0.611 (0.255–1.460)	0.265
Thrombocytopenia [*n* (%)]	7 (8.0)	8 (21.6)	4.499	3.153 (1.050–9.469)	0.034*
Coagulation abnormalities [*n* (%)]	24 (27.6)	15 (40.5)	2.021	1.790 (0.799–4.012)	0.155

PAIS, perinatal arterial ischemic stroke; PHS, perinatal hemorrhagic stroke; OR, odds ratio. $\bar{x}\pm s$.

*P* < 0.05 indicates statistical significance.

Postnatal risk factors: Among the neonates with cerebral infarction, 51 (41.1%) experienced postnatal infections, including 3 confirmed cases of purulent meningitis. Specific pathogens were identified in 11 instances: 4 Mycoplasma infections, 4 enterovirus infections, 2 Group B Streptococcus infections, and 1 case of cytomegalovirus infection. In the PAIS group, infections were reported in 36 cases (41.4%), compared to 15 cases (40.5%) in the PHS group. Congenital heart defects were present in 39 cases (31.5%), including 10 isolated cases of patent ductus arteriosus (PDA). Complications related to PDA included pulmonary hypertension (7 cases), atrial septal defect (1 case), pericardial effusion (3 cases), coarctation of the aorta (1 case), ventricular septal thickening (2 cases), simple atrial septal defect (5 cases), ventricular septal defect (2 cases), arrhythmia (1 case), and simple pulmonary hypertension (7 cases). Hypercoagulable states were observed in 9 cases (7.3%), with a distribution of 7 cases (8%) in the PAIS group and 2 cases (5.4%) in the PHS group. Thrombocytopenia was present in 15 cases (11.7%), including 7 cases (8%) in the PAIS group and 8 cases (21.6%) in the PHS group; coagulation abnormalities were noted in 39 cases (31.5%), with 24 cases (27.6%) from the PAIS group and 15 cases (40.5%) from the PHS group (Table 2).

Statistical comparisons of risk factors between the PAIS and PHS groups indicated significant differences in conversion from failed trial labor to cesarean section and thrombocytopenia between hemorrhagic and ischemic strokes (*P* < 0.05). Specifically, the conversion from a failed trial of labor to cesarean section was significantly more common in the PAIS group compared to the PHS group (*P* = 0.012). Additionally, there was a notable association between postnatal thrombocytopenia and the PHS group compared to the PAIS group (*P* = 0.034). There were no significant differences in other risk factors between the two groups (*P* > 0.05) (Table 2).

### Clinical manifestations and EEG findings

Among the 124 neonates included in the study, 107 (86.3%) were admitted within three days of birth, with a median age at onset of symptoms being 2 days. The earliest onset was observed immediately after birth, while the latest occurred at 27 days post-birth. Clonic seizures were reported in 78 cases (62.9%), which included unilateral limb clonic seizures (contralateral to the lesion) in 22 cases (28.2%) and generalized tonic or limb clonic seizures in 56 cases (71.8%). The average onset of clonic seizures was at 4 days, with a duration averaging 50.77 ± 57 h (ranging from 2 to 432 h). In addition, 17 cases (13.7%) primarily presented with apnea, while 18 cases (14.5%) were admitted mainly for jaundice. Cyanosis was the predominant symptom in 7 cases (5.6%), and there were also reports of 1 case of tachycardia, 2 cases of vomiting, and 1 case with no apparent clinical symptoms. At admission, 53 patients (42.7%) exhibited incomplete elicitation of primitive reflexes. Muscle tone abnormalities were noted in 62 cases (50%), with decreased muscle tone in 24 cases (19.3%) and increased muscle tone in 38 cases (30.6%). A total of 114 patients (91.9%) underwent a well-established electroencephalogram, and 54.4% (62/114) exhibited abnormal EEG activity. Among them, there were 23 cases of electro-clinical (with clinical sign) (accounting for 37.1% of abnormal EEG activity), 18 cases of electrographic only (without clinical signs) (accounting for 29% of abnormal EEG activity), 21 cases of unclassified seizure type (accounting for 33.9% of abnormal EEG activity), and 52 cases of no abnormal discharges, accounting for 45.6% (52/114). There was no significant difference between the clinical manifestations between the PAIS and PHS groups (*P* > 0.05) (Table 3).

**Table 3:** Comparison of the clinical manifestations between the PAIS and PHS groups.

Variable	PAIS group (*n* = 87)	PHS group (*n* = 37)	*χ*^2^/*Z* value	OR (95% CI)	*P*-value
Onset time [M (P25, P75), h]	24 (10, 41)	12 (2.3, 53.5)	1.037	—	0.300
Seizures [*n* (%)]	58 (66.7)	27 (73)	0.479	1.35 (0.576–3.163)	0.489
Apnea [*n* (%)]	11 (12.6)	6 (16.2)	0.280	1.337 (0.455–3.933)	0.597
Jaundice [*n* (%)]	10 (11.5)	8 (21.6)	2.146	2.124 (0.764–5.909)	0.143
Cyanosis [*n* (%)]	5 (5.7)	2 (5.4)	0.006	0.937 (0.173–5.063)	0.94
Abnormal primitive reflexes [*n* (%)]	39 (44.8)	14 (37.8)	0.518	0.749 (0.341–1.646)	0.472
Muscle tone abnormalities [*n* (%)]	42 (48.3)	20 (54.1)	0.347	1.261 (0.583–2.726)	0.556
EEG abnormalities [*n* (%)]	42 (52.5)^a^	20 (58.8)^b^	0.385	1.293 (0.574–2.911)	0.535
Electro-clinical (with clinical sign)	18 (22.5)	5 (14.7)			
Electrographic only (without clinical signs)	9 (11.3)	9 (26.5)			
Unclassified seizure type	15 (18.8)	6 (17.6)			

EEG, electroencephalograms; PAIS, perinatal arterial ischemic stroke; PHS, perinatal hemorrhagic stroke; OR, odds ratio. $\bar{x}\pm s$.

^a^ Indicates that the total number of patients was only 80, of which EEG was not done in 7 patients.

^b^ Indicates that the total number of patients was only 34, of which EEG was not done in 3 patients.

### Magnetic resonance imaging (MRI) lesion location and prognosis analysis

All 124 neonates underwent comprehensive cranial MRI and diffusion-weighted imaging (DWI) scans to confirm the diagnosis of cerebral infarction, with 87 cases (70.2%) in the PAIS group and 37 cases (29.8%) in the PHS group. Lesion localization showed that 53 cases (41.4%) had lesions in the left cerebral hemisphere, 31 cases (24.2%) had lesions in the right hemisphere, and bilateral lesions were observed in 44 cases (34.4%). Additionally, lesions affecting the thalamus and basal ganglia were identified in 33 cases (25.8%).

Of the total cohort, 9 neonates succumbed to severe illness or had treatment withdrawn due to family concerns regarding prognosis. Follow-up was conducted for the remaining 115 cases through outpatient visits and telephone inquiries; however, 56 were lost to follow-up due to changes in contact information or refusal to participate, resulting in a follow-up cohort of 59 cases. The longest follow-up extended until age 5 years, during which 11 cases developed adverse outcomes such as cerebral palsy, hemiplegia, and epilepsy. During the hospitalization of these children, the EEG showed significant low voltage (more than 50% amplitude reduction) on the affected side or even the whole brain, frequent polymorphic epileptic waves, multifocal spikes, sharp waves, and paroxysmal high-amplitude slow waves, etc., and the NMR results showed a combination of thalamus and basal ganglia injuries. Comparative analysis of MRI lesion locations and prognoses between the PAIS and PHS groups indicated no significant differences in clinical outcomes (*P* > 0.05) (Table 4).

**Table 4:** Comparison of MRI lesion location and prognosis between the PAIS and PHS groups.

Variable	PAIS group (*n* = 87)	PHS group (*n* = 37)	*χ*^2^/*Z* value	OR (95% CI)	*P*-value
Left cerebral hemisphere lesions [*n* (%)]	39 (44.8)	14 (37.8)	0.518	0.749 (0.341–1.646)	0.472
Right cerebral hemisphere lesions [*n* (%)]	21 (24.1)	10 (27)	0.116	1.164 (0.485–2.795)	0.734
Bilateral cerebral hemisphere lesions [*n* (%)]	32 (36.8)	12 (32.4)	0.214	0.825 (0.365–1.863)	0.643
Thalamic and basal ganglia lesions [*n* (%)]	21 (24.1)	12 (32.4)	0.914	1.509 (0.648–3.514)	0.339
Adverse outcomes [*n* (%)]	7 (15.6)	4 (28.6)	1.193	2.171 (0.529–8.914)	0.275
Cerebral palsy [*n* (%)]	2 (28.6)^a^	1 (25)^b^			
Severe hemiplegia [*n* (%)]	1 (14.3)^a^	2 (50)^b^			
Partial limitation of motor function [*n* (%)]	4 (57.1)^a^	1 (25)^b^			
Seizures [*n* (%)]	2 (28.6)^a^	2 (50)^b^			

PAIS, perinatal arterial ischemic stroke; PHS, perinatal hemorrhagic stroke; OR, odds ratio. $\bar{x}\pm s$.

^a^ Number of cases 7.

^b^ Number of cases 4.

Of the 59 children followed up, the longest current follow-up was up to 5 years of age, with a total of 11 children experiencing adverse outcomes. EEG during hospitalization showed significant low voltage (amplitude reduction of more than 50%) on the affected side or even the whole brain, multiple polymorphic epileptic waves, multifocal spikes, sharp waves, and paroxysmal high-amplitude slow waves, and MRI results showed composite damage to the thalamus and basal ganglia regions. Among them, 7 cases in the arterial ischemic infarction group had neurological complications of varying degrees, 5 cases had infarction of the main trunk of the middle cerebral artery, and 2 cases had infarction of the middle cerebral artery below the M1 segment and the cortical branch; 2 cases had cerebral palsy, 1 case had severe hemiparesis, 4 cases had partial limitation of motor function, and 2 cases had residual epileptic seizures. In the hemorrhagic infarction group, 4 cases had neurological complications, 2 cases had seizures, 1 case had cerebral palsy, 2 cases had hemiparesis, and 1 case had partial limitation of motor function. Comparative analysis of MRI lesion location and prognosis in the PAIS and PHS groups showed no clinically significant differences (*P* > 0.05) (Table 4).

## Discussion

Recent advancements in imaging technology have significantly improved the diagnostic rate of PCI, and considerable research has been conducted on the etiology and pathogenesis of PCI. Studies have indicated that PAIS accounts for most lesions in neonatal cerebral infarctions (13). In this study, there were 87 cases (70.2%) patients presented with PAIS, a proportion similar to that reported in the literature (14). Research has shown that PCI occurs more frequently in males, with a male-to-female ratio of 1.3–1.6:1 (15). Risk factors for PAIS include multiple pregnancies, chorioamnionitis during pregnancy, preeclampsia, diabetes, autoimmune diseases, thrombotic diseases, perinatal infections, perinatal asphyxia, intrauterine distress, infants with small gestational age, abnormalities in amniotic fluid, umbilical cord, placenta, emergency cesarean delivery, postnatal infections, bacterial meningitis, congenital heart disease, hypoglycemia, polycythemia, and disseminated intravascular coagulation (DIC) (16–19). In this study, male neonates accounted for 66.9% of all cases, which is significantly higher than female neonates. However, no significant differences were observed between the PAIS and PHS groups, suggesting that sex did not affect the different outcomes of cerebral stroke.

In analyzing previously reported risk factors, comparisons between the two groups revealed statistically significant differences in certain factors, including failed labor trials resulting in cesarean delivery and thrombocytopenia. The PHS group had a notably lower rate of cesarean delivery compared to the PAIS group, suggesting that natural childbirth increases the risk of birth trauma, which has been associated with hemorrhagic stroke in some studies (20), although direct evidence is still lacking and further confirmation is required from larger sample studies. Moreover, a higher proportion of neonates in the PHS group exhibited postnatal thrombocytopenia. In a study by Bruno et al. of 42 full-term and late preterm infants with hemorrhagic stroke, 16 (38%) exhibited one or more coagulation disorders, 13 of which were associated with thrombocytopenia (21). Thrombocytopenia has been reported to be common in hemorrhagic stroke and can lead to intracranial hemorrhage and death, which is consistent with our findings (22). Thrombocytopenia is more likely to be a clinical manifestation of hemorrhagic infarction, and the cause may be due to bleeding or secondary to DIC, resulting in platelet depletion.

The majority of studies on perinatal cerebral infarction have focused on the risk factors and prognosis of arterial ischemic infarction, and the risk factors of hemorrhagic infarction have not yet been reported in large samples and multicenters, and some studies (21–23) have mentioned that hemorrhagic infarction has been associated with protein C and protein S deficiencies, maternal immune disorders, and thrombosis. In the present study, there was a case of hemorrhagic infarction in a child who developed hemorrhagic infarction at a later stage of life after reporting maternal prenatal systemic lupus erythematosus. This suggests that hemorrhagic infarction may be correlated with maternal immune diseases, but the sample size is too small and further large-sample studies are needed. In recent years, a new foreign study reported that COL4A1 gene mutation was associated with perinatal intracranial hemorrhage, suggesting that hemorrhagic infarction may be related to genetic factors (24). With the popularization of genetic testing, genetic factors will become a new research direction for risk factors of hemorrhagic infarction.

In both the PAIS and PHS groups, seizures were the most common initial symptom, with nearly half of the neonates exhibiting abnormalities in muscle tone and primitive reflexes, consistent with the findings reported in the literature (25). EEG abnormalities were present in approximately 50% of the cases in both groups, with no significant differences between groups. In the PIS group, apnea was more frequently observed as the initial symptom, whereas jaundice was more common in the PHS group, and it was associated with an increase in unconjugated bilirubin levels following intracranial hemorrhage.

Cranial MRI, including standard MRI, diffusion-weighted imaging (DWI), magnetic resonance angiography (MRA), and magnetic resonance venography (MRV), is the gold standard for diagnosing neurological cerebral infarction (NCI). Typically, within 1 week of onset, lesions show low signal on T1-weighted images (T1WI) and high signal on T2-weighted images (T2WI). After one week, the affected areas had high signal intensities on T1WIs and low signal intensities on T2WIs. Early lesions are frequently located in the left cerebral hemisphere, particularly in areas supplied by the middle cerebral artery (14, 26). This study's results are similar, with both groups predominantly showing lesions in the left cerebral hemisphere. There were no significant differences in the location of brain lesions between the two groups. However, the PHS group had more lesions involving the thalamus and basal ganglia and a higher proportion of poor outcomes, possibly due to hemorrhagic strokes that typically involve more extensive and broader areas of damage.

Most of the current studies on cerebral infarction in neonates do not differentiate between preterm and term infants, and only a few studies have reported cerebral infarction in preterm infants. In a German surveillance study, a significantly higher incidence was reported in preterm infants, 32/100.000 in preterm infants and 21/100.000 in term infants (27). Furthermore, multiple regression analysis showed a 1.57-fold increased risk of PAIS in preterm infants compared to term infants, and the study attributed the higher risk of preterm birth to intrauterine hypoxia and birth asphyxia, but there were no differences in risk factors between preterm and term infants (17). Our study also did not differentiate between preterm and term infants, but there was no significant difference in the rates of intrauterine distress and birth asphyxia between the two groups in our study, which was considered to have no significant effect on clinical outcomes.

This study was a retrospective analysis conducted at a single center. To achieve an adequate sample size, cases were collected over an extended period. In earlier cases, MRI scans often did not include MRA and MRV, which complicated the classification of PCI subtypes based on MRI findings, particularly in distinguishing between hemorrhagic and ischemic lesions. Although we made adjustments based on onset time, lesion location, and affected areas, some residual confounding bias persisted due to cases where differentiation was not feasible. Additionally, the prolonged timeframe for assessing the prognosis of neonatal cerebral infarction resulted in a significant loss to follow-up, primarily due to changes in contact information and inconsistent follow-up practices. Moreover, the outcomes obtained through telephone follow-ups may have been influenced by parental bias, which limits the reliability of these assessments and hinders more comprehensive investigations. To address these challenges, we have established a specialized referral and follow-up system in collaboration with pediatric health and rehabilitation departments to enhance the reliability of prognostic research for related conditions.

## Conclusions

This comparative study highlights significant differences in clinical characteristics and risk factors between PAIS and PHS. Our findings indicate that PAIS is more prevalent within the studied population, with a notable correlation between failed labor trials resulting in cesarean sections and the incidence of PAIS. This suggests a potential link between complications during labor and the occurrence of ischemic strokes. In contrast, postnatal thrombocytopenia was found to be significantly more common in the PHS group, indicating a possible association between low platelet counts and hemorrhagic strokes. These findings enhance our understanding of the distinct risk profiles associated with PAIS and PHS, paving the way for future research aimed at developing targeted preventive and interventional strategies for each stroke subtype. Further prospective studies are needed to validate these associations and investigate additional potential risk factors.

## Data Availability

The original contributions presented in the study are included in the article/Supplementary Material, further inquiries can be directed to the corresponding authors.

## References

[1] Dunbar M Kirton A. Perinatal stroke: mechanisms, management, and outcomes of early cerebrovascular brain injury. Lancet Child Adolesc Health. (2018) 2(9):666–76. 10.1016/S2352-4642(18)30173-1 30119760

[2] Raju TN Nelson KB Ferriero D Lynch JK, NICHD-NINDS Perinatal Stroke Workshop Participants. Ischemic perinatal stroke: summary of a workshop sponsored by the National Institute of Child Health and Human Development and the National Institute of Neurological Disorders and Stroke. Pediatrics. (2007) 120:609–16. 10.1542/peds.2007-0336 17766535

[3] Govaert P Ramenghi L Taal R de Vries L Deveber G. Diagnosis of perinatal stroke: I. Definitions, differential diagnosis and registration. Acta Paediatr. (2009) 98:1556–67. 10.1111/j.1651-2227.2009.01461.x 19663912

[4] Martinez-Biarge M Ferriero DM Cowan FM. Perinatal arterial ischemic stroke. Handb Clin Neurol. (2019) 162:239–66. 10.1016/B978-0-444-64029-1.00011-4 31324313

[5] Clive B Vincer M Ahmad T Khan N Afifi J El-Naggar W. Epidemiology of neonatal stroke: a populationbased study. Paediatr Child Health. (2020) 25(1):20–5. 10.1093/pch/pxy194 33390736 PMC7757779

[6] Kirton A Armstrong-Wells J Chang T Deveber G Rivkin MJ Hernandez M Symptomatic neonatal arterial ischemic stroke: the international pediatric stroke study. Pediatrics. (2011) 128(6):e1402–10. 10.1542/peds.2011-1148 22123886

[7] Baddouh N Elbakri S Draiss G Mouaffak Y Rada N Younous S. Cerebral venous thrombosis in children: a series of 12 cases. Pan Afr Med J. (2019) 32:1–8. 10.11604/pamj.2019.32.22.17656 PMC6522156 31143327

[8] Caspar-Teuscher M Studer M Regényi M Steinlin M Grunt S, Swiss Neuropediatric Stroke Registry Group. Health-related quality of life and manual ability 5 years after neonatal ischemic stroke. Eur J Paediatr Neurol. (2019) 23(5):716–22. 10.1016/j.ejpn.2019.08.002 31473077

[9] Loo S Ilves P Mannamaa M Laugesaar R Loorits D Tomberg T Long-term neurodevelopmental outcome after perinatal arterial ischemic stroke and periventricular venous infarction. Eur J Paediatr Neurol. (2018) 22(6):1006–15. 10.1016/j.ejpn.2018.07.005 30249407

[10] Kwok TC Dineen RA Whitehouse W Lynn RM McSweeney N. Don Sharkey* Neonatal stroke surveillance study protocol in the United Kingdom and Republic of Ireland. Open Med. (2022) 17:1417–24. 10.1515/med-2022-0554 PMC9449691 36128449

[11] Pressler RM Cilio MR Mizrahi EM Moshé SL Nunes ML Plouin P The ILAE classification of seizures and the epilepsies: modification for seizures in the neonate. Position paper by the ILAE task force on neonatal seizures. Epilepsia. (2021) 62(3):615–28. 10.1111/epi.16815 33522601

[12] Tsuchida TN Wusthoff CJ Shellhaas RA Abend NS Hahn CD Sullivan JE American Clinical neurophysiology society standardized EEG terminology and categorization for the description of continuous EEG monitoring in neonates: report of the American clinical neurophysiology society critical care monitoring committee. J Clin Neurophysiol. (2013) 30(2):161–73. 10.1097/WNP.0b013e3182872b24 23545767

[13] Ferriero DM Fullerton HJ Bernard TJ Billinghurst L Daniels SR DeBaun MR Management of stroke in neonates and children: a scientific statement from the American Heart Association/American Stroke Association. Stroke. (2019) 50(3):e51–96. 10.1161/STR.0000000000000183 30686119

[14] Lehman LL Rivkin MJ. Perinatal arterial ischemic stroke: presentation, risk factors, evaluation, and outcome. Pediatr Neurol. (2014) 51(6):760–8. 10.1016/j.pediatrneurol.2014.07.031 25444092

[15] Villapol S Faivre V Joshi P Moretti R Besson VC Charriaut-Marlangue C. Early sex differences in immune-inflammatory responses to neonatal ischemic stroke. Int J Mol Sci. (2019) 20(15):3809. 10.3390/ijms20153809 31382688 PMC6695584

[16] Rattani A Lim J Mistry AM Prablek MA Roth SG Jordan LC Incidence of epilepsy and associated risk factors among perinatal ischemic stroke survivors. Pediatr Neurol. (2019) 90:44–55. 10.1016/j.pediatrneurol.2018.08.025 30409458

[17] Sorg AL von Kries R Klemme M Gerstl L Weinberger R Beyerlein A Risk factors for perinatal arterial ischemic stroke: a large case-control study. Dev Med Child Neurol. (2020) 62(4):513–20. 10.1111/dmcn.14347 31489622

[18] Miranda B Fonseca AC Ferro JM. Patent foramen ovale and stroke. J Neurol. (2018) 265(8):1943–9. 10.1007/s00415-018-8865-0 29680895

[19] Darmency-Stamboul V Chantegret C Ferdynus C Mejean N Durand C Sagot P Antenatal factors associated with perinatal arterial ischemic stroke. Stroke. (2012) 43(9):2307–12. 10.1161/STROKEAHA.111.642181 22738921

[20] Martinez-Biarge M Cheong JLY Diez-Sebastian J Mercuri E Dubowitz LMS Cowan FM. Risk factors for neonatal arterial ischemic stroke: importance of the intrapartum period. J Pediatr. (2016) 173:62–8. 10.1016/j.jpeds.2016.02.064 27049002

[21] Bruno CJ Beslow LA Witmer CM Vossough A Jordan LC Zelonis S Hemorrhagic stroke in term and late preterm neonates. Arch Dis Child Fetal Neonatal Ed. (2014) 99:F48–53. 10.1136/archdischild-2013-304068 23995383 PMC3864979

[22] Lee CC Lin JJ Lin KL Lim WH Hsu KH Hsu JF Manifestations, outcomes, and etiologies of perinatal stroke in Taiwan: comparisons between ischemic, and hemorrhagic stroke based on 10-year experience in A single institute. Pediatr Neurol. (2017) 58:270–7. 10.1016/j.pedneo.2016.07.005 28087259

[23] Wang J-J Shi K-L Li J-W Jiang LQ Caspi O Fang F Risk factors for arterial ischemic and hemorrhagic stroke in childhood. Pediatr Neurol. (2009):40:277–81. 10.1016/j.pediatrneurol.2008.11.002 19302940

[24] Bersani I Ronci S Savarese I Piersigilli F Micalizzi A Maddaloni C COL4A1 gene mutations and perinatal intracranial hemorrhage in neonates: case reports and literature review. Front. Pediatr. (2024) 12:1417873. 10.3389/fped.2024.1417873 38978838 PMC11228817

[25] Dunbar M Mineyko A Hill M Hodge J Floer A Kirton A. Population-based birth prevalence of disease-specific perinatal stroke. Pediatrics. (2020) 146(5):e2020013201. 10.1542/peds.2020-013201 33115795

[26] Harteman JC Groenendaal F Kwee A Welsing PM Benders MJ de Vries LS. Risk factors for perinatal arterial ischemic stroke in full-term infants: a case-control study. Arch Dis Child Fetal Neonatal Ed. (2012) 97(6):F411–6. 10.1136/archdischild-2011-300973 22445901

[27] Sorg AL von Kries R Klemme M Gerstl L Felderhoff-Muser U Dzietko M. Incidence estimates of perinatal arterial ischemic stroke in preterm- and term-born infants: a national capture-recapture calculation corrected surveillance study. Neonatology. (2021) 118(6):727–33. 10.1159/000514922 33794541

